# Impact of goal orientation on moral competence development in youth

**DOI:** 10.1038/s41598-024-74697-7

**Published:** 2024-10-09

**Authors:** Mateusz Ludwiczak, Małgorzata Bronikowska

**Affiliations:** Department of Recreation, Poznan University of Physical Education, Królowej Jadwigi 27/39 St., 61-871 Poznan, Poland

**Keywords:** Psychology, Human behaviour

## Abstract

The study had two aims. The first was to investigate correlation between specific types of goal orientation as a predictor of prosocial and antisocial functioning and level of moral competences. The second, was to verify whether goal orientation has an impact on the development of moral competences of students participating in a pedagogical intervention based on non-linear pedagogy. Task and Ego Orientation in Sport Questionnaire and Moral Competence Test were used to assess 468 secondary school students aged 15 in goal orientation and moral competences level. There was no correlation between the level of task and ego orientation and the level of moral competence. Under the influence of pedagogical experiment students with high level of task orientation achieved a statistically significantly higher change in the level of moral competences (η^2^ = 0.04). Study suggests that high level of task orientation is more susceptible to the development of moral competencies. Also, employing a non-linear pedagogy model in physical education, provides a conducive environment for the development of moral competences and should be implemented in the training of future physical education teachers.

**Supplementary Information:**

The online version contains supplementary material available at 10.1038/s41598-024-74697-7.

## Introduction

Physical education (PE) as a school subject possesses the potential to address diverse areas of knowledge, cognitive functions, behavior, and skill development among school students^1–4^. PE lessons facilitating social skills development and cultivation through physical activity (PA) opportunities^5^. Unlike other subjects within the school environment, PE classes significantly impact student interactions due to the inherent structure of the lessons (practical lesson with active participation) fostering enhanced adaptability^6,7^. A crucial aspect of the instructional process involves establishing a conducive climate and attitude towards activity implementation^8^. Nicholls’ Achievement Goal Theory (AGT)^9^ as a theoretical framework of this study suggests that the motivation and performance are perceived based on students’ evaluation of their abilities and competence. Furthermore, Duda^10^ proposes considering two motivational orientations: task orientation and ego orientation. Task-oriented individuals perceive success as an opportunity for personal growth, primarily driven by intrinsic motivation^11^. In contrast, ego-oriented individuals focus on outcome comparisons with others^12^. As abovementioned, task orientation is closely tied to intrinsic motivation, which produces positive emotions stemming from task engagement, such as enjoyment and pleasure. The degree of intrinsic motivation exhibited by individuals predicts their persistence and performance in the environments in which they operate^13–17^. In PE, task-oriented activities are associated with effort, group cohesion, enjoyment, persistence in learning, and other positive variables at a high level^18^. On the other hand, ego-oriented individuals are perceived as competitive characters whose behaviors are driven by personal gains: the relentless pursuit of victory or minimal effort to achieve success in PE classes^19^. The predispositions related to orientation (ego-/task) are also influenced by the individual’s demonstrated level of moral competencies^20^.

Moral competence, recognized as a fundamental dimension of human functioning^21^, plays a vital role in guiding decision-making and judgment. It entails the ability to address problems and conflicts using moral principles as a compass, relying on discussion and deliberation rather than resorting to violence^22^. Within the school environment, the moral dimension finds expression through moral education, and PE emerges as a subject that offers opportunities for experiencing and practicing moral development within the curriculum^23^. Parisi and colleagues^24^ emphasize the significance of motivation and highlight evidence pointing to a correlation between the level of moral competence and ego- and task-related orientations. Specifically, a positive correlation occurs between the level of moral competence and a high score of task orientation, and at the same time a positive correlation is observed between a high ego index and a low level of moral competence^24^. Within the domain of sports, a correlation between moral behavior and goal orientation has been established^25^. Previous studies have confirmed that athletes with higher level of moral competences and prosocial behavior are more frequently task-oriented and exhibit greater adherence to sportsmanship compared to ego-oriented athletes^26^. Additionally, research has indicated that a task-oriented motivational climate fostered in PE classes promotes moral development among elementary school children^27^, which may suggest that introducing carefully prepared content with a specific focus on task-oriented activities during PE classes from an early age may potentially lead to positive changes in children’s attitudes. It is worth emphasizing that pedagogical interventions undertaken in the context of motivational profile have also shown potential in enhancing goal-oriented motivation among youth^18,28,29^. Within the domain of PE, only a limited number of targeted interventions have thus far been formulated with the aim of cultivating the moral and social dimensions of students^27,30,31^. These programs are usually based on models such as Teaching Personal and Social Responsibility^32^ and/or Sport Education^33^, which focus on achieving predetermined outcomes. Another noteworthy approach to apply as an element in the developmental program might be the model of Non-linear Pedagogy (NP), which adopts a student-centered approach to a learning process^34^. When effectively implemented through well-structured physical education (PE) classes, NP addresses three core psychological needs—autonomy, competence, and relatedness—and has been shown to foster higher levels of intrinsic motivation in students compared to traditional PE lessons^11^. Additionally, NP is strongly rooted in Self-Determination Theory^35^, enabling it to create an environment conducive to positive learning experiences. Unlike traditional direct instruction, NP is based on guided discovery, i.e. enabling students the search for solutions by experiencing and manipulating constraints by the teacher, giving the student autonomy in action and assessing his or her behavior based on the achievement of the goal and not on the technic of execution^36^. It is also important to note that according to AGT, task and ego goal orientations are considered as an orthogonal dimensions^37^ in the context of PE^38^. This means that individuals can exhibit high or low levels of either orientation independently, and can also have varying levels of both simultaneously.

In view of the abovementioned, and considering the aforementioned results of prior research, the primary objective of our study is to scrutinize whether specific types of orientation (ego-task) exhibited by the examined youth delineate variations in their levels of moral competencies. Building upon the findings of previous investigations^24^ and taking into consideration an orthogonal dimensions of task and ego orientations, a hypothesis can be ventured that the group displaying increased level of task orientation coupled with a diminished manifestation of ego orientation is likely to manifest a higher level of moral competencies compared to the students who demonstrate elevated ego orientation and reduced task orientation. The second aim of the study is to examine which type of orientation—ego or task—is more likely to influence the development of moral competence as a result of the pedagogical experiment.

Based on the above considerations, it can also be hypothesized that students with a high level of task orientation and a low level of ego orientation will be characterized by the greatest change (increase) in moral competence.

## Materials and methods

### Participants

The study was conducted during the school years 2021/2022 and 2022/2023, and it comprised two distinct stages. In the initial phase of the study a questionnaire survey was administered to secondary school students (who had just started their education at this school as first-year high school students), to assess their level of moral competence and motivational orientation (i.e., ego and task). The sample assessment was performed considering sample size and power. To calculate the sample size needed to detect a correlation with effect size 0.3, significance level α = 0.05, with the statistical test power of 0.8 a total of at least 84 participants are required (calculations were made using the G-Power program). A total of 468 secondary school students (15 years old) participated in the study. School students were recruited from one of the secondary public schools, being a vocational school within a city of 600.000 habitants. The selected school adheres to standards and policies that are consistent with those of other institutions within the urban area of the metropolitan city. The exclusion criteria incorporated secondary school students who did not complete the consent form, did not attend, or complete the questionnaire. Due to the lack of significant gender differences in the context of moral development^39–41^ and the research tools used, the participants were not distinguished by gender. Additionally, previous interventions conducted in the PE environment did not reveal any discrepancies in moral reasoning between boys and girls^27^. Instead of gender, they were distributed into four groups based on their orientation level (by scores they gained; see the tool protocol below). Hence, the first group (G1) comprised individuals with low ego and low task orientation (*n* = 155), the second group (G2) consisted of those with low ego and high task orientation (*n* = 112), the third group (G3) included individuals with high ego and low task orientation (*n* = 90), and the fourth group (G4) encompassed those with high ego and high task orientation (*n* = 111).

The second phase of the research involved implementing a self-designed pedagogical experiment. This decision was driven by previous scientific reports wherein such methods of pedagogical intervention were employed and yielded positive outcomes^27^ regarding the main aim of fostering the development of moral competences through participation in PE classes. For the second stage conventionally average effect size for the social sciences (partial η2 = 0.06) with the statistical test power of 0.8; for two groups and two measurements, to demonstrate the interaction effect “repeated measurements x goal orientation” at the significance level α = 0.05, a total of at least 128 participants are required (calculations were made using the G-Power program). In our study a total of 354, aged 15 from first year high school participants from the same school were assigned in number 177 students to the experimental group (EG) with intervention and the same number (*n* = 177) to the control group (CG) with standard PE classes. The number of participants in the control and experimental groups was determined by the fact that the allocation was dictated by the students’ class attendance (usually a class has about thirty students) and classes were randomly assigned to both groups. Each group consisted of students from six classes. The exclusion criteria in this stage were the same as in the first stage, additionally excluded students who had year-round sick leave and did not participate in PE classes. Comparison of groups (experimental and control) in terms of characteristics (ego and task level) indicated no differences between groups. In experimental group the division was as follows: the first group (G1) comprised individuals with low ego and low task orientation (*n* = 60), the second group (G2) consisted of those with low ego and high task orientation (*n* = 52), the third group (G3) included individuals with high ego and low task orientation (*n* = 25), and the fourth group (G4) encompassed those with high ego and high task orientation (*n* = 40). The study protocol was approved by the Bioethics Committee of the Karol Marcinkowski Medical University in Poznan (Poznan, Poland; N. 117/21).

Tools.

#### Task and ego orientation in sport questionnaire (TEOSQ)

The Polish adaptation of the *Task and Ego Orientation in Sport Questionnaire* (TEOSQ), as described by Tomczak and colleagues^42^, was utilized in the study. It worth mentioning that the TEOSQ tool was developed for utilization in both PE and sports contexts, serving as a versatile resource for enhancing performance and fostering holistic development^42,43^. This is actually a scale, which comprises 13 items, with 7 items relating to task orientation issues (e.g. “I learn a new skill, and it makes me want to practice more”) and 6 items pertaining to ego orientation issues (e.g. “I am the only one who can do the play or skill”). Participants were required to indicate the extent to which each statement applied to them on a 5-point Likert scale, ranging from 1 (strongly disagree) to 5 (strongly agree). Cronbach’s Alpha for the ego subscale was 0.84, and for the task subscale 0.81^42^. The use of the tool allowed the participants to be divided into four groups consecutively (see above). The division was determined using the median split method. Score for task orientation (28), where scores equal to or below were categorized as low, and scores above were classified as high. Similarly, for ego orientation, the median score was 19, with scores equal to or below classified as low, and scores above categorized as high.

#### Moral competence test (MCT**)**

To account for cultural factors, the Polish validated and certified version of the Moral Competence Test^44^ (MCT), adapted by Nowak^45^, was employed. This test includes two real-life dilemmas. Each story has twelve statements (six in favor and six against the proposed behavior). Students responded on a nine-point Likert-type scale, from − 4 (totally disagree) to + 4 (totally agree) and agree or disagree with the statements, corresponding to one of the six stages of moral development as outlined by Kohlberg’s theory^1^. Cronbach’s Alpha for the scale was 0.80. According to empirical research on moral judgment, four basic assumptions must be met for the MJT to be considered valid and reliable^46^. These are: preference hierarchy, quasi-simplex structure, cognitive-affective parallelism, and correlation with education level. The above criteria were all met in the Polish version of the MJT as it was indicated by the relevant study. The collected data was subsequently used to calculate the C-index, which serves as an indicator of the individual’s level of moral competence. The C-index score ranges from 1 to 100 and reflects the individual’s ability to evaluate arguments based on moral characteristic. It quantifies the degree to which an individual’s personal judgments and reasoning are influenced by moral concerns and principles, as opposed to personal opinions and constructs. C-index scores below 9 are considered very low, scores below 19 are classified as low, scores between 19 and 29 are regarded as medium, scores above 29 to 39 are deemed high, and scores above 39 are categorized as very high in regard of moral competency.

### Intervention design and implementation

The intervention was based on NP model^34^ and encompassed competitive tasks designed to elicit behaviors associated with coping in high-emotional-load situations, wherein students were exposed to both the experience of defeat and the experience of victory (see Supplementary Table). The implementation of the planned intervention in the context of PE was approved by the principal and the School Board of Education at the selected school, allowing for research to be conducted among a participant pool of over 140 individuals. The PE teachers at the school underwent training by an expert with appropriate knowledge and pedagogical experience in the intervention, and they were provided with well-prepared structured lesson plans and additionally with all necessary didactic and educational materials. Moreover, before the experiment teachers had the opportunity to observe the activities included in the intervention content during demonstration lessons.

The fidelity of intervention’s implementation was determined by observation, using O’Donnell’s^47^ criteria for assessing fidelity, focusing on duration (the frequency of program delivery), adherence (whether program components were delivered as prescribed), and quality of delivery (how well the program material was implemented). Teachers followed the intervention program (see Supplementary Table), and confirmation of compliance of the content with the required level was confirmed by observation. Changes in order of tasks performed within the moral stage were allowed, considering school year calendar, weather conditions and school infrastructure. The intervention protocol employed in this study was devised following the prevailing didactical guidelines in the field of PE. Each session (45 min) was structured into three distinct phases – introductory/warming up, main part of the lesson, and cooling down/conclusions – incorporating principles of NP and tailored to the age-appropriate level of moral development exhibited by the participants. Whereas the observation protocol was conducted by a person with high experience in the pedagogical process and scientific experience in the field of research in didactics of PA. There were no significant deviations in the way the intervention was conducted.

The research was launched at the end of August with participants’ recruitment, and in early September, all participants completed a pre-test to establish the initial state of the particular group’s moral competence level for further analysis.

The implemented PE program consisted of twenty-six sessions throughout the entire school year. The basic structure of the intervention comprised three components: warm-up (10–12 min), main part of the lesson (25–30 min), cool-down, and brief summary with a discussion for further thoughts and reflection (5–7 min). Each class lasted 45 min and took place in the school’s sports gym and/or school sport’s fields. The intervention program encompassed various forms of PA carefully planned by the PE teacher, designed to introduce real-life socio-cognitive dilemmas during their execution. These dilemmas prompted students to engage in critical thinking, reflection, and the exchange of viewpoints (interpersonal communication). All of this was aimed at fostering deliberate decision-making within the experimental group. In this respect students had the opportunity to face situations that required them to make decisions regarding adherence to game rules, group dynamic principles, the desire to win at any cost, and the ability to trust others in the context of competition or cooperation (including team-building and problem-solving activities with communication and intra-/interpersonal skills, etc.). A crucial part of each lesson was the summary phase, during which students could express their own reflections on their initial assumptions and final behavior during the lesson. The program aimed to create an environment in which students progressed through all stages of structural development theory^1^, enabling them to resolve emerging dilemmas during the lesson. Each student reacted in their own way to the emerging dilemmas and had the opportunity to contemplate them during the final reflection immediately after the class (during discussion part of the class). After the intervention was completed, there was a second term (i.e., post-test) for the control (CG) and experimental group (EG). At the same time as the intervention for the experimental group (EG) took place, the control group (CG) completed physical education classes in accordance with the curriculum based on direct instruction. The classes included plays and games, team sports and other activities. The difference was that the activities were not modified to occur planned moral dilemmas during the lesson in which students made decisions based on their moral competence, the teacher’s attention was mainly focused on the teaching of technical skills. The summary of the classes also did not contain open questions regarding moral decisions made during the classes related to reflection.

### Statistics

First, the descriptive statistics which concerned all study participants (*n* = 468) (arithmetic mean (M), standard deviation (SD), were calculated, the distribution of variables (normality control – W-Shapiro Wilka and Levene’s test). Subsequently a correlation analysis was conducted, consisting in examining whether the level of moral competence is related to the level of goal orientation (i.e., ego, task) and after that multiple regression in which the dependent variable was the level of moral competence and the independent variables were ego and task. Both statistical calculations showed no statistical significance in the sample of participants (*p* > 0.05).

Next, actions related to the typology of the ego and task levels were taken. Due to the orthogonal dimension of the factors, four types had been distinguished: (1) low ego and low task orientation, (2) low ego and high task orientation, (3) high ego and low task orientation, (4) high ego and high task orientation, to compare the divided groups, analysis of variance was used.

To verify the impact of the intervention on the level of moral competence (increase in the level of moral competence) two-way analysis of variance (the first factor – a group, the second factor – repeated measurement) was used. The control (CG) and experimental (EG) group were compared with the same number of participants (*n* = 177). Participants of the CG were randomly selected from students (the same environment) who took part in initial phase of research. Then they were traditionally conducted PE classes during the experiment period.

To assess whether the change under the influence of the intervention depends on task and ego, a 3-way analysis of variance was used in the intervention group.

All statistical analyses were performed using TIBCO Statistica version 13.3 (TIBCO Software Inc., Palo Alto, California, USA). More information about additional statistical calculations can be found in Appendix B.

## Results

### Initial analyses

The correlation between the level of task and ego orientation and the level of moral competence was examined. There was no statistically significant correlation between the indicated factors (Ego *p* > 0.05, *r*=− 0.023; Task *p* > 0.05, *r* = 0.01). There was no statistically significant regression model for moral competences, which included ego and task, r^2^ = 0, F(2.465) = 0.177. For ego β = 0.026; *p* > 0,05. For task β = 0.01; *p* > 0,05.

Then, the difference in moral competences was analyzed in groups distinguished according to task and ego types. There were no statistically significant differences between the groups F(3,464) = 0.799, *p* > 0.05.

Table 1 presents descriptive statistics of four groups who took a part in experiment in the context of C-index (Mean ± SD) as the final score referring to the individual’s ability to assess an argument based on their moral quality. Additionally, the table provides the number of students in each group (N) and the standard deviation (SD).

**Table 1:** Typological division of research participants by task and ego orientation and their level of moral competence.

Group	*N*	M C-index	SD
G 1	155	15.0	14.9
G 2	112	17.1	14.7
G 3	90	14.8	11.7
G 4	111	14.5	13.0

### Intervention’s analyses

**Fig. 1:**
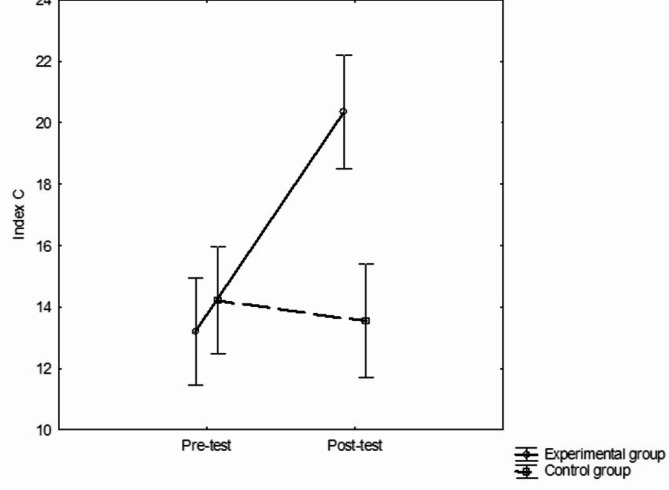
Description of changes under the influence of pedagogical experiment in two terms. Pre-test and Post-test for experimental group (EG) and control group (CG).

Devoted to intervention noticeable was a statistically significant interaction effect in factors group x repeated measurement F(1, 352) = 31,553, *p*=0.001; η^2^ = 0.08. In the experimental group (EG), the post-test assessment showed a statistically significant increase in moral competence compared to the pre-test (HSD Tukey: *p* < 0.005). On the other hand, in the control group (CG), no statistically significant difference was obtained between the values of the moral competence level in the post-test and the pre-test (HSD Tukey: *p* > 0.05) (see Fig. 1).

To verify the relationship between the factors of goal orientation and moral competences in changes under pedagogical experiment, a 3-way ANOVA was conducted. Table 2 presents result of assessment whether the change under the influence of the intervention (increase of moral competences) depends on task and ego orientation.

**Table 2:** Results of 3-way ANOVA with factors: repeated measure x ego x task.

Effect	SS	DF	MS	F	*p*	η^2^	λ	α = 0.05
	86991.5	1	86991.5	419.5	0.0001	0.70	419.5	1.000
Ego	7.2	1	7.3	0.03	0.85	0.00	0.03	0.053
Task	15.2	1	15.3	0.07	0.78	0.00	0.07	0.058
Ego × task	23.8	1	23.8	0.12	0.73	0.00	0.11	0.063
Error	35871.4	173	207.35					
Repeated measure	2988.3	1	2988.3	34.3	0.0001	0.16	34.3	0.999
Repeated measure × ego	78.9	1	79.0	0.9	0.342	0.01	0.9	0.157
Repeated measure × task*	620.6	**1**	**620**.**6**	**7**.**1**	**0**.**008**	**0**.**04**	**7**.**1**	**0**.**756**
Repeated measure × ego × task	100.7	1	100.8	1.1	0.283	0.01	1.1	0.187
Error	15062.6	173	87.1					

*Notes statistical significance *p* < 0.05.

Significant values are in bold.

**Fig. 2:**
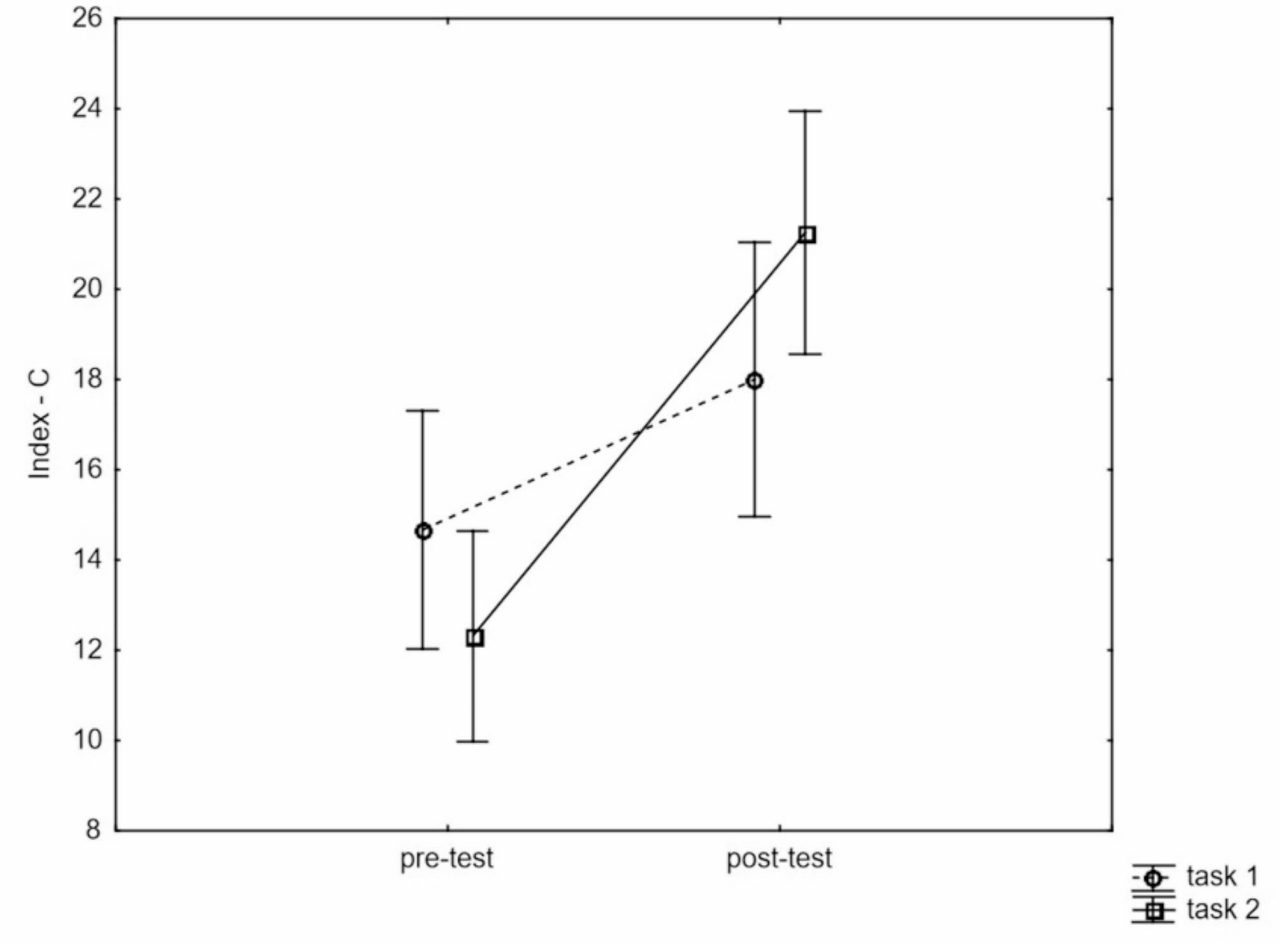
Description of changes under the influence of pedagogical experiment in two terms. Pre-test, post-test for group with low level of task (task 1) and high level of task (task 2).

There was not a statistically significant interaction effect repeated measure x ego orientation level x task orientation level (*p* > 0.05) observed. There was a statistically significant interaction effect task orientation x repeated measurement F(1, 173) = 7,1279, *p* = 0,01; η^2^ = 0,04. In the group with high level of task orientation the level of moral competence in the post-test was statistically significantly higher than in the pre-test (HSD Tukey: *p* < 0.01). Whereas, in the group with low level of task orientation no statistically significant difference was obtained between the values of moral competence level in the post-test and the pre-test (HSD Tukey: *p* > 0.05). (see Fig. 2).

The level of ego orientation does not determine changes in moral competences in relation to the high level of task orientation. Both for low ego level (G1) and high ego level (G2). Participants who presented a high task-oriented level significantly increased their level of moral competence through the intervention, while among participants with a low task-oriented level (G3 and G4) there were no statistically significant changes in the level of moral competence before and after the pedagogically designed intervention.

## Discussion

The first objective of the study was to examine whether specific types of ego and task orientation are associated with differences in moral competence. The results of this study indicated that the level of moral competence was not related to the motivational orientation of the participants, encompassing both high and low levels of ego and task orientations. These findings shed new light on the understanding of ego orientation in the context of PE. Previous study^24^ suggested, that individuals with a high level of ego orientation were often associated with lower moral competence. Other studies^48^ have also shown that activities characterized by ego-oriented motivation, such as table soccer games, are linked to more antisocial behaviors compared to those driven by task-oriented motivation. In the field of sports, performance (ego-oriented) goals (both approach and avoidance) have shown a negative association with the C-index, which defines level of moral competence. However, it is important to note that the correlation between performance goals and moral competence is positive, but low, below 0.3. Conversely, mastery (task-oriented) goals (both approach and avoidance) have not exhibited a significant correlation with moral competence in previous studies^49^. Whereas in Proios and colleagues^25^ study reviled a weak positive correlation (below 0.3) between task orientation and stages 2, 4, and 6 of moral reasoning. Furthermore, there are few reports indicating that a high-performance orientation is associated with lower moral acting and can serve as an indicator of athletes’ antisocial judgment and behavior^49,50^. Based on our research findings on school students, they did not show statistical differentiation in terms of moral competence based on their levels of ego and task orientations. However, it is important to emphasize that individuals with a high level of ego orientation are often focused on competition^19^. Nonetheless, this does not directly imply non-compliance with the rules governing the game. It is also noteworthy that in our study, individuals with a high level of ego orientation and a low level of task orientation did not exhibit statistically significant differences compared to other types of orientation.

The second aim of our research related to intervention was to investigate whether specific types of ego-task relationships are predisposed to the development of moral competences under the influence of a pedagogical experiment based on non-linear pedagogy. According to Mouratidou and colleagues^27^ a task orientation is more beneficial to the development of moral competence as such. This finding aligns with our results, which demonstrated a significant relationship between task orientation level and the development of moral competence. Students with a high level of task orientation exhibited statistically significant increase in moral competence compared to those with a low task orientation level. The research suggests the potential for task development during PE lessons under the influence of intervention. Additionally, our study indicates that motivational orientation is a factor influencing changes in the level of moral competence. On the other hand, the level of ego orientation did not have an impact on the level of moral competence, which is an intriguing result contradicting the previous findings of Parisi and colleagues^24^, who have demonstrated a correlation between high ego orientation and low moral competence.

Our research also confirmed that pedagogical intervention during PE lessons, which incorporated elements of competition, goal striving, cooperation, and the search for compromises, provided an opportunity to develop various types of goal orientation among young people. This implies that professionally designed teaching process can support the development of moral competences regardless of students’ goal orientation, enabling them to enhance this aspect. However, this requires primarily appropriate subject matter preparation as well as professional experience from PE teachers. In addition, their attitude towards the profession is important, with special attention given to collaborating with other individuals, in this case children and adolescents who are in a continuous process of holistic development. But as Bronikowski^51^ explains young adolescents need sense of coherence (of understanding) to maintain their engagement in PA, but also to pay attention to the moral standards that are often neglected by PE teachers and thus are not considered as important in sports. It might be the problem which is related to the MCL they present quality of professional training of PE teachers as role models themselves^23^. Moreover, according to Chen and Ennis^52^ and others^23,53,54^, PE teachers still present the “disciplinary mastery” orientation focusing on developing performance proficiency in sport skills mostly and understanding of performance-related knowledge. And previous studies have shown that moral development is positively correlated with task orientation^10,55,56^, indicating that interventions promoting a task-oriented motivational climate could be beneficial for moral development. Other authors have observed that interventions in PE classes aimed at improving motivation led to a significant decrease in the perception of a performance climate^18^. The stimulation of motivational PE classes climate to enhance students’ task orientation has also been explored^57^. Overall, our research demonstrates, that all experimental groups, which were a part of the designed intervention achieved statistically significant improvements comparing to the control group. However, it can be inferred that students with a high level of task orientation may be more effective in developing their moral competences within the PE environment. Nevertheless, the above presented results indicate that the implementation of a properly prepared pedagogical intervention based on established theories can yield tangible positive outcomes.

### Study limitations

The research was conducted in one school, which may affect the level of representativeness of the group. This means that the sample is not an ideal example of a representative group of students with individual goal orientations compared to a potential representative population sample. However, the researchers decided to proceed with the study despite this limitation, considering the uniform educational system and upbringing methods in Polish schools, and the fact that the school does not differ in its local environment from other schools in the metropolitan area. Additionally, it is worth mentioning that obtaining consent from school authorities or parents to conduct research in public schools has become increasingly difficult in the last decade. It is worth considering the use of the median splits as statistical methods. Although this method is widely used in psychology and has some advantages, it also has its limitations. However, due to the lack of possibility of substantively dividing the groups, we used an empirical (arbitrary) division using the method related to the median and classification into low and high indicators with the division of participants into groups. Another limitation can be considered the Moral competence Test used for measuring the level of moral competence. This tool, which evaluates level, is a cognitive test (pen and paper), whereas changes visible in results in the moral dimension are based on activities (behaviors, decision making, reactions, emotions). However, there is no other test better yet that can assess moral competence through procedural knowledge. Nonetheless a real dilemma, as a method of evaluating moral values based on hypothetical rather than theoretical dilemmas, can be of great environmental importance because participants can provide relevant situational content in relation to their everyday life and the cultural context that they experience.

## Conclusion

The findings of the study indicate that specific types of ego and task orientation do not statistically differ in terms of the level of moral competence. However, in the context of the implemented pedagogical intervention, which focuses on the development of moral competences and is based on non-linear pedagogy the level of task orientation does have an impact on positive changes in moral competence. Students with a high level of task orientation demonstrated a statistically significant increase in the level of moral competence compared to participants with a low level of task orientation. Interestingly, the level of ego orientation did not influence changes in moral competence. These results suggest that well organized and conducted PE process, utilizing a non-linear pedagogy model, provides a conducive environment for the development of moral competences across all types of goal orientation. These studies demonstrate the importance of an innovative approach to preparing future educators for the process of teaching PE. We therefore recommend reviewing the academic curriculum to include non-linear pedagogical approaches in the context of physical education for young people.

## Electronic supplementary material

Below is the link to the electronic supplementary material.

Supplementary Material 1

Supplementary Material 2

## Data Availability

All raw data are available on request to the corresponding author.
