# Supplementary material for: Impact of goal orientation on moral competence development in youth
Source: Sci Rep. 2024 Oct 9;14:23578. doi: 10.1038/s41598-024-74697-7 (PMC11464530; doi:10.1038/s41598-024-74697-7)
Supplement: Supplementary file 1 — Supplementary Material 1 [file 41598_2024_74697_MOESM1_ESM.docx]

| **Session** | **Aspect of development** | **Moral stage** | **Lesson content** | **Moral dielmma/details for teachers** |
| --- | --- | --- | --- | --- |
| 1 | **Follow the rules** | 1 | **Game with ringo** (modification of ringo’s number).  The task of the participants is to pass the ringo to the person who holds the gymnastic stick in a specific place on the pitch. Players can't run with a ringo. When the ringo falls to the ground, the opposing team takes over the ringo. | The student in the game decides whether to behave according to the rules or take advantage of the situation when no one is watching (when there are several ringos in play, participants' attention is distracted) and take the ringo (which fell down), obtaining an advantage that is against the rules. |
| 2 | **Follow the rules** | 1 | **Game with tennis balls**.  The participants are divided into several groups. All but one group are "guardians", whose aim is to protect their own tennis ball (everyone has a ball) which is near them on ground. Designated group tries to collect all the tennis balls within the given time (2 minutes). Each group has time (round) to collect balls. | The guards should have their eyes closed. The participant decides whether he/she follows the rules or tries to catch the person who tries to take the ball at all costs. |
| 3 | **Equipment sharing / outcome relationship decisions** | 1 | **Basketball skills in task** (shooting/free throws). Participants divided into small groups. For two minutes, the task is to score as many points as possible (free throws). Each group has only one ball, the first person in a row decides whether to throw or pass the ball to the next person. Baskets are ranked from best (number 1) to worst (number 5).  Promotions and relegations take place after two minutes. The person who has been promoted (e.g., move from basket number 4 to number 3) starts at the next basket last, the person who has been relegated (e.g., move from basket number 2 to number 3) starts first. | The decision whether the student will share the equipment, giving everyone an equal chance to compete. Will there be internal rules created by the group? Will participants adapt to mutually agreed rules? |
| 4 | **Equipment sharing / outcome relationship decisions** | 1 | **Volleyball skills in game**  Students get into specific groups, then try to make as many passes as possible using both upper and lower hands for three minutes. Then, each individual counts the passes scored after each subsequent round. | Students choose themselves into teams of 2, 4, 6, 8 players (the number increases with each subsequent round),  the question is whether they will join the team because of their skills or relations with other participants. Will each of the participants have the opportunity to contact the ball, will they have the same opportunity to improve their skills and gain points? |
| 5 | **Assessment of own abilities** | 2 | **Task with time control**  Students have 90 seconds to complete the activity. Before starting the task, they determine the estimated number of repetitions of a given exercise (during the task, they cannot stop practicing even if they reach the assumed number). After 90 seconds, if they get more repetitions than expected, they deduct one point for each repetition. For example when player assumed 40 repetitions and he did 60, at the end of the round he gets 20 points, if he did 70 he gets 10 points.  Tasks to do: Squats, push-ups, jumps, burpees. | Everyone independently assess abilities related to the task (activity), defining own constraints. Counts own points. This is to show the ability to assess their own abilities, whether the student overestimates or underestimates their abilities. It is also worth paying attention to the attitude of the participants. |
| 6 | **Assessment of own abilities** | 2 | **Dodgeball with extra tasks.**  Before game (round lasts 5 minutes) each participant chooses one of the four possible sashes (colour). Each sash gives you the opportunity to earn extra points. Strength - throwing a fitness ball directly to the person in the base; Speed - touch the end line of the opponent's field and return to your own field of play (being not hit by opposite team);Agility - avoid being hit throughout the round; Dexterity - catching the ball. After round player can change the colour of sashe. | Each participant can assess their motor skills, which is their advantage and how they can help the team. Each participant counts own points (this is not verified by the teacher afterwards).  Each subsequent lesson assumes more and more independence and responsibility on the part of the student, making them co-creators of the learning process. |
| 7 | **Responsibility for others** | 2 | **Tag for three teams.**  The team is assigned a colour. Each team is running from one colour and chasing another colour. The caught person freezes in place. The team with the most active people after a certain time (2 minutes) wins. The winning team in the first three rounds assigns a movement task to the other two teams. In the next two rounds, the winning team gives a task to all teams (even themselves). | Observation of reaction whether the team, after winning, will give a task that can be completed by the others whether if the task is to be completed by all teams, it will be a similar task or easier.  Paying attention to the behaviour when we have control over others - whether we are reasonable towards them and are able to manage them.  If we lost in previous rounds, do we want to make others suffer? |
| 8 | **Responsibility for others** | 2 | **Submarines**  Several people stand in a row to form a submarine. Only the last person in the row has his eyes open and is the captain. The boat moves on the march and is steered by a tap on the shoulder passed as a signal to the next people. Tap on the right shoulder - turn the boat to the right by 90 degrees, similarly to the left. A tap on both arms means launching a torpedo, which is the first person in the row – he/she can take 15 steps alone and then he/she returns to the end of the row and he/she takes over the role of captain. A point is awarded for hitting another submarine with a torpedo. | Responsibility for the other participants (leadership), their health, not to hit obstacles - a wall, another boat. Ability to make decisions under time and space pressure.  It is important that each person can act as the captain of the submarine. |
| 9 | **Empathy / support for others** | 3 | **Task with functional activities**  The class is divided into groups. The task is to hit a specific target (e.g., goal) one after another in the row using their own footwear. Then the groups have time to consult whose shoes belong to whom and in a row race they try to find the shoes of the person behind them. Then they help tie their shoes and run to the end of the row. | The task requires communication with other participants, team support and a positive attitude towards the task. Participants compete in groups, therefore failure may result from team disproportions and this may determine the final result.  Will students see competition through the prism of skill?  Will they hold grudges against other people from the team?  Will they cope with the pressure of time? |
| 10 | **Empathy / support for others** | 3 | **Two teams facing each other**.  People from the team hold hands, forming a "chain" of hands. The goal is to push the opposing team over the line or break the "chain" from the opposing team's hands. | It is worth paying attention to whether the students adapt the strategy to their abilities. They change the position of the team due to the possibilities, they take into account the emotions of other students. It is worth noting that people with high body weight have a significant impact on the final effect. It is worth emphasizing that every person is important during PE lessons. |
| 11 | **Cooperation** | 3 | **Task in 3 teams**  Participants divided into teams. The task of the team is to create one letter or number chosen by the teacher in a given round, creating it from their own bodies lying on the ground. | Participants could decide how create the structure of task. Will students communicate each other in task? Is it visible leader in team? Whether some people's ideas are rejected by the group or considered. Teacher can make competition by adding a score for the speed of completing the task. |
| 12 | **Cooperation** | 3 | Task in one team  The task is carried out in one large group. The task is to create a specific shape e.g., square, triangle with a rope (each participant holds a rope) while keeping their eyes closed.  “Gordian Knot” - Participants form a circle of at least 5 participants and approach each other, then close their eyes and raise their hands. They take turns catching other people's free hands (first right hand and then from other person left one), so that each person has the opportunity to contact the other participant's hands. Then the participants open their eyes and their task is to solve/loosen the "Gordian Knot" without breaking it. | Participants could decide how create the structure of task. Will students communicate each other during the task? Is there a visible leader in the team? Whether some people's ideas are rejected by the group or considered. |
| 13 | **Critical / Independent thinking** | 4 | **Basketball skills in task** (passing, dribbling)  The task consists of basketball skills: passing, dribbling, throwing to the basket. Participants have to perform specific behaviours in individual places on the pitch, what matters is maintaining the intensity of the task, where each participant will be involved in the task. | Can the participant break out of the pattern of thinking? If player is able to take into account the organization of the entire team. If student is only concerned with his task or observes the need for the team and being able to adapt for situation on the pitch. |
| 14 | **Critical / Independent thinking** | 4 | **Task in 4 teams**  Participants are divided into teams. Two teams face each other on a certain line. The game is turn based. The task is carried out by one person from a specific team. Student task is to run up to the opposing team standing on the line, touch the person from the opposing team who is trying to catch the fleeing person at that moment. Student get a point for returning to his team. In case of being caught by the chaser, the escaping player changes the team. | whether the person chooses the easiest opponent or takes up the challenge and competes with people with similar or higher motor control potential. In conclusion, it is worth asking what motivated people to choose an easier or more difficult opponent. |
| 15 | **Universality of physical activity** | 4 | **Play in pairs**  Group are divided into two teams. Each team lines up. The competition takes place in pairs opposite each other. One team is number “one” the other team is number “two”. On the command “one” person from the first team runs away and the person from the second team chases. At the command "two" analogous situation. After round the winning person goes to a duel with the person who lost the duel in the pair in front, the losing person goes to the duel with the person who won the duel behind.  Math problems can then be added as a form of choosing a fleeing and chasing person. If the result is odd, “one” escapes, if the result is even, “two” escapes. Tasks can be related to various mathematical operations. | Indicating the universality of PE classes, showing that it is not only physical activity, but other skills are also important. Showing the possibility of assessing the situation and potential of a person from different perspectives. |
| 16 | **Universality of physical activity** | 4 | **Game with 3 teams - "Bricklayer**"  Participants divided into three teams.One of the participants is choosen as a brickleyer. The teacher behind the "bricklayer" shows which team has immunity - even if it is caught by the bricklayer, it continues to play and does not become a "brick". | Observation of the behaviour of those who have immunity in a given round.  Teacher can point out that, even if you have immunity, it's worth acting like a regular player, not making it easier for the bricklayer, because in the next round you may not have immunity and because of the behaviour from the previous round, you may have a harder task - more bricks in the wall. It is worth comparing this behaviour to other situations in life. |
| 17 | **Conflict resolution** | 5 | **Game Tic-Tac-Toe - with 6 teams** (3 boards).  The students judge and decide who won the duel. Then they switch board depending on who won and who lost. | Encourage students to be able to resolve conflicts. Do they justify their decisions, behaviours? Do they engage in discussions individually or in groups, or there are people in the group who do not engage in dialogue? |
| 18 | **Conflict resolution** | 5 | **Task with a changing number of teams**  The task is to walk a certain length using the given number of arms and legs | Students choose themselves into teams of 2, 4, 6, 8 players (the number increases with each subsequent round).  Joint search for solutions in a situation under time pressure. Paying attention to whether students make an effort of their own idea or suggest solutions from other groups. |
| 19 | **Finding common compromise** | 5 | **Game with teams** (depending on how many people in the class).  One person is inside a hula hoop. The rest of the group shapes the circle 1 meter from the cicle. The aim is to go beyond the hula hoop touching the person outside and get back into the hula hoop before someone else steps in hula hoop faster. A person from the centre has to go with both feet outside the hula hoop. | Students decide howthe game will look like. Will they focus on competition, scoring or winning at all costs? A person who finds themselves inside the hula hoop can hold the game, and sometimes make it unplayable e.g., afraid to lose their place inside or by scoring one point and staying in the middle to wait for the end of the round and not give others a chance to score. Will the students find a common solution to the situation? How will it be received by the group, what emotions will accompany the participants during the summary of the lesson? |
| 20 | **Finding common compromise** | 5 | **Task in pairs**  Students pair up. They turn their backs to each other, a cone on the ground between them. The teacher gives warm-up tasks to perform, at the same time saying that at a certain signal players should catch the cone. Whoever gets the cone gets a point. The teacher deliberately gives different signals and then announces that no one scores, asking each time if someone hasn’t bent down to pick up the cone. | The task is to show the difference between hearing information and listening and absorbing information. Paying attention to observing individual situations not only from one's own perspective and focusing on selective listening. |
| 21 | **Trusting others** | 6 | **Volleyball skills in small-sided games**  Games 2v2 or 3v3 on very small pitches. Player can use both high and low passes. | Deciding whether the student will focus on scoring (counting points), even though the assigned person will be responsible for this. |
| 22 | **Trusting others** | 6 | **Task with various equipment and in teams** **– obstacle** **course**  Students choose their own equipment needed to create an obstacle course then they will guide participants who will keep their eyes closed and rely only on auditory information from the creator of the obstacle course. | Are students able to delegate responsibility to others, trust in the performance of tasks by others? It is worth asking students what emotions accompanied them when they relied solely on a specific person, whether there were individuals they did not trust or had a limited sense of security? |
| 23 | **Creating the rules** | 6 | **Play without equipment**  Tag, in which students jointly determine the rules by adding various modifications. | Joint determination of the rules of the game by the participants, the teacher only gives the background. Verification whether all participants follow the common rules, whether the rules are developed by everyone or passed on by only a few people. |
| 24 | **Creating the rules** | 6 | **Game with 2 teams without equipment**  A game without equipment, which consists of running across the opponent’s half without being caught (a point is scored), if someone is caught in the first round is out. The teacher sets only the time of the first round (2 minutes) and then allows the students to modify all the rules of the game. | Joint determination of the rules of the game by the participants, the teacher only gives the background. Verification whether all participants follow the common rules, whether the rules are developed by everyone or passed on by only a few people. |
| 25 | **Creating the rules** | 6 | **Game with 2 teams with hula-hoops**  A palant/baseball-based game using hula hoops as bases. The teacher allows the students to modify the rules of the game. | Joint determination of the rules of the game by the participants, the teacher only gives the background. Verification whether all participants follow the common rules, whether the rules are developed by everyone or passed on by only a few people. |
| 26 | **Creating the rules** | 6 | **Game with balls** (volleyball, basketball, tennis)  The aim of the game is to make as many passes as possible with specific balls (i.e., volleyball, basketball, tennis). Students establish rules related to the way the ball is passed, scoring, playing space, additional rules related to the takeover of the ball by the other team. | Joint determination of the rules of the game by the participants, the teacher only gives the background. Verification whether all participants agree with and follow the common rules, whether the rules are developed by everyone or passed on by only a few people. |
