# Supplementary material for: Impact of goal orientation on moral competence development in youth
Source: Sci Rep. 2024 Oct 9;14:23578. doi: 10.1038/s41598-024-74697-7 (PMC11464530; doi:10.1038/s41598-024-74697-7)
Supplement: Supplementary file 2 — Supplementary Material 2 [file 41598_2024_74697_MOESM2_ESM.pdf]

Additional information and clarification about statistical calculations used in the article

To enhance the clarity and precision of above prepared research, here is a more refined version of your statement, focusing on the essential statistical calculations information.

In this appendix, we present a comprehensive overview of the statistical analyses conducted in the course of this study. The primary objective of this section is to provide a transparent and detailed account of all relevant calculations, ensuring that any methodological concerns or questions arising from the article can be addressed with clarity. This focused approach highlights only the most significant statistical procedures, avoiding extraneous information, to maintain a clear understanding of the methods and results discussed in the main text. In short: this version emphasizes transparency and precision, keeping the focus on key calculations.

Due to the lack of possibility of substantively dividing the groups, we used an empirical (arbitrary) division using the method related to the median (median split method) and classification into low and high indicators with the division of participants into groups, which at the same time did not exclude the possibility of interaction of two factors (repeated measurement x task) or (repeated measurement x ego) to verify the influence of one type of orientation on changes in moral competences. We know the advantages and disadvantages of median split method however, this is a method that is commonly used in psychological research. We also conducted a cluster analysis k-means for cases. The obtained results did not provide an unambiguous interpretation, after consulting with a statistician, we ultimately chose the median split method, although we mentioned it in the limitations.

In the analysis of the second aim, we used a division by group using discontinuous variable. Please note that two types are defined in the Figure 2: task 1 and task 2. In the statistical calculations, participants were coded for their level of orientation defined in the ego and task factors. The number one indicated low level of orientation, the number two indicated high level

of orientation, so the division could show changes in both low- and high-level of participants' orientation.

Also in relation to second aim of the study experimental and control group were similar in terms of ego and task orientation. We did not find significant differences based on the comparative analysis of task: t=1,15, df=352, p=0,25. For Ego there was a marginally significant: t=-2,03, df=352, p=0,043. However, this was mainly due to the large group size. The effect sizes (Cohen's D) for the differences were small. For ego = 0.22, for task = -0.12

Additionally, having data from post-test term and we checked task orientation and ego after the intervention. There were no differences between terms inside group. We also have date to compare control (CG) and experimental group (EG), there was no interaction effect indicating that there were different changes in groups. Repeated measure (pre – post) x task: $F(1, 352)=3{,}69$, $p>0{,}05$, partial $\eta^2=0.01$. Repeated measure (pre – post) x ego: $F(1, 352)=0{,}12$, $p>0{,}05$, partial $\eta^2=0.01$.
